# Progress and Prospects for a Nucleic Acid Screening Test Set

**DOI:** 10.1089/apb.2023.0033

**Published:** 2024-09-18

**Authors:** Nicole E. Wheeler, Craig Bartling, Sarah R. Carter, Adam Clore, James Diggans, Kevin Flyangolts, Bryan T. Gemler, Brittany Rife Magalis, Jacob Beal

**Affiliations:** ^1^ Institute of Microbiology and Infection, University of Birmingham, Birmingham, UK.; ^2^ Battelle Memorial Institute, Columbus, Ohio, USA.; ^3^ Science Policy Consulting LLC, Arlington, Virginia, USA.; ^4^ Integrated DNA Technologies Inc., Coralville, Iowa, USA.; ^5^ Twist Bioscience Corporation, South San Francisco, California, USA.; ^6^ Aclid Inc, New York, New York, USA.; ^7^ Department of Pathology, Immunology and Laboratory Medicine, University of Florida, Gainesville, Florida, USA.; ^8^ Raytheon BBN Technologies, Arlington, Virginia, USA.

**Keywords:** biosecurity, standards, nucleic acid synthesis, synthetic DNA, export controls, Australia group

## Abstract

**Objective:**

DNA synthesis companies screen orders to detect controlled sequences with misuse risks. Assessing screening accuracy is challenging owing to the breadth of biological risks and ambiguities in risk definitions. Here, we detail an International Gene Synthesis Consortium working group’s rationale and process to develop a prototype DNA synthesis screening test dataset, aiming to establish a baseline of screening system accuracy to compare with various screening approaches.

**Methodology:**

Construction of the prototype test dataset involved four tool developers screening nucleic acid sequences from three taxonomic clusters of controlled organisms (*Orbivirus*, *Francisella tularensis*, and *Coccidioides*). Results were mapped onto predefined, comparable categories, checking for consensus or conflicts. Conflicts were grouped based on gene annotation and resolved through discussion.

**Results:**

The process highlighted several long-standing challenges in DNA synthesis screening, including the qualitative differences in approaches taken by screening tools. Our findings highlight the lack of clarity in assessing pathogen sequences with respect to regulatory control language, compounded by scientific uncertainty. We illustrate the current degree of consensus and existing challenges using classification statistics and specific examples.

**Conclusions and Next Steps:**

This prototype underscores the necessity of expert-regulator coordination in assessing gene-associated risks, offering a template for creating test sets across all taxonomic groups on international control lists. Expanding the working group would enrich dataset comprehensiveness, enabling a transition from species-focused to function-focused regulatory controls. This sets the foundation for quality control, certification, and improved risk assessment in DNA synthesis screening.

## Introduction

The burgeoning field of synthetic genomics is revolutionizing biomedicine and biotechnology research, driving a rapidly increasing demand for custom-made synthetic DNA sequences.^1^ As the technology required for designing, synthesizing, and assembling nucleic acids improves, the ability to cost-effectively acquire large amounts of biologically relevant synthetic nucleotides continues to improve as well.^2^ Fast, high-volume availability of synthetic nucleotides is critical for the growth of the bioeconomy but, as a dual-use technology, may be subject to potential misuse, whether accidental or intentional. There are broad multilateral regulatory frameworks governing export control of potentially dangerous DNA sequences, but these generally offer few details on how a DNA synthesis provider can pragmatically determine whether a DNA sequence is, in fact, dangerous and/or regulated. Although export control does not necessarily imply control over domestic usage, in practice, the coordinated export control lists typically define a superset of the collection of dual-use organisms that are also controlled by domestic regulations. Similarly, the U.S. government has published guidance^3,4^ for synthesis providers to screen nucleic acid sequences prior to manufacture, but these rules and guidelines leave most technical decisions regarding how to implement screening open to interpretation.

As a result, current screening systems are highly heterogeneous, with most still being ad hoc systems implemented by individual synthesis companies for their own proprietary internal usage. More recently, several organizations have built commercial sequence screening tools,^5–10^ which are now being used by some DNA synthesis companies. All of these tools utilize distinct algorithms and reference databases to assess sequence risks and inform synthetic nucleic acid providers of the existence of potentially dangerous and/or controlled sequences in an order. The ambiguity in screening guidance and differences between screening tool implementations creates a need for a common screening test suite against which to measure tools to ensure the robustness of nucleic acid screening implementations and to identify ambiguities in need of further clarification by regulatory authorities.

Building such a test suite extends beyond the capability of any one individual organization, instead requiring a collaborative effort across diverse domains, including virology, microbial and fungal pathogenesis, protein biophysics, bioinformatics, software engineering, and business workflow management. This collaborative ethos is vital to nucleic acid screening, where shared efforts can streamline advancements, accelerate progress, and reduce both risks and administrative burdens. The development of such a test set will also have broader benefits: in the United States, the National Institute for Standards and Technology (NIST) and other agencies have been tapped in the recent “Executive Order on the Safe, Secure, and Trustworthy Development and Use of Artificial Intelligence”^14^ to help establish technical resources for effective screening, and similar efforts are being contemplated in the United Kingdom and the European Union (EU). Datasets that can be used to assess the accuracy of screening will serve as key elements of these technical resources.

At its core, a properly designed test suite should create the ability to measure whether a screening system meets a minimal set of requirements for efficacy as well as to compare screening tools with one another. This empirical assessment can provide invaluable insights into the comparative performance of diverse screening systems. Such comparisons allow for establishing minimum standards for acceptable performance, refining existing methodologies, and steering the evolution of screening technologies toward enhanced effectiveness. Rigorous testing and comparisons between systems will be critical as synthesis providers seek to develop and implement “best practices” that go beyond baseline screening capabilities, for example, by flagging a wider range of potentially harmful sequences, as called for in updated guidance from the U.S. government.^4^

A standardized test suite can also significantly enhance the defensibility of decisions made during the screening process. This pertains particularly to the determination of whether or not an order merits human review. Standardization can bring clarity and consistency to decision-making, strengthening the overall reliability and integrity of the screening outcomes and the uniformity of those outcomes across the global nucleic acid synthesis industry.

**Figure 1.**
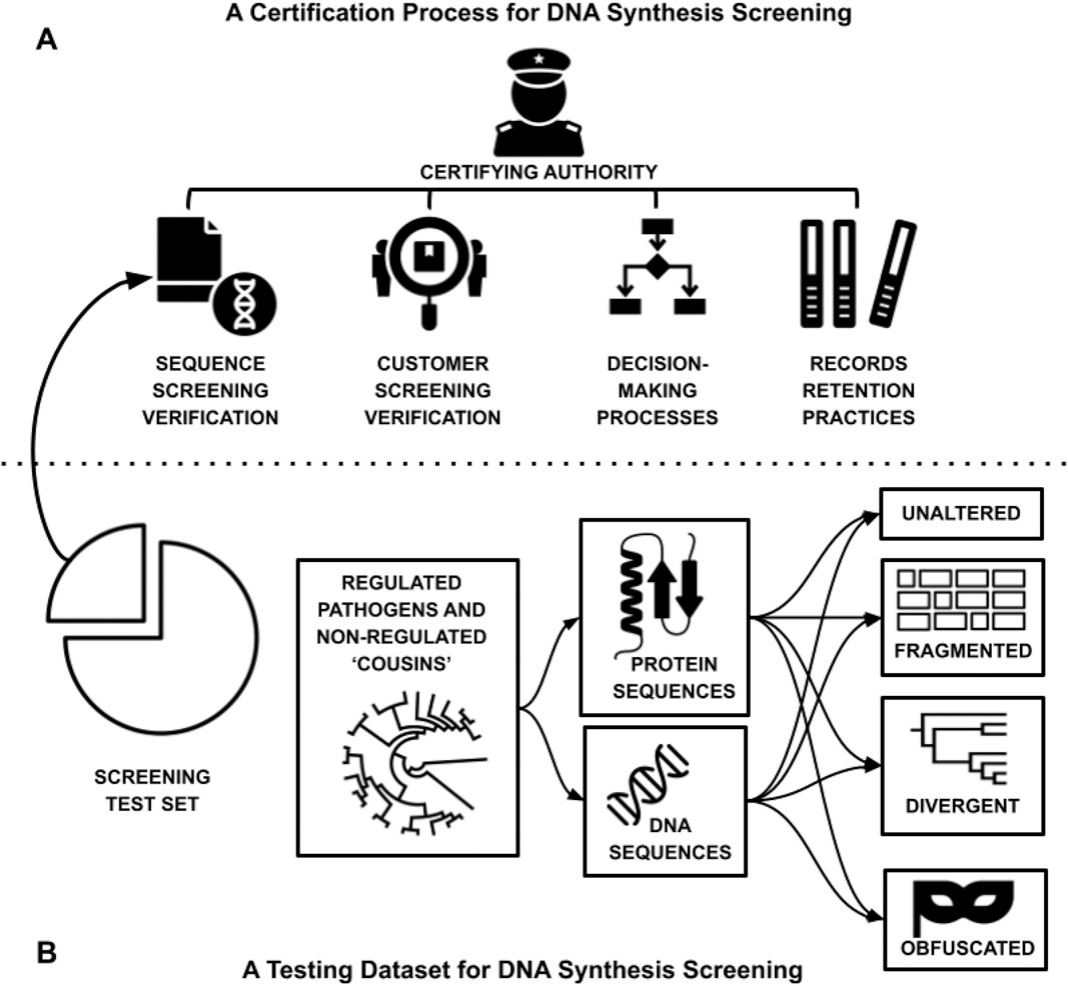
Components of a potential nucleic acid screening certification process and its relation to a screening test set.

Finally, it is imperative to distinguish the purpose of a test suite for measuring screening performance from specific test data that might be used in the certification of a screening tool’s performance. These serve distinct but complementary purposes. A certification process for sequence screening would require a certifying authority—and no such authority exists at present. A certification process would also encompass broader aspects beyond just sequence screening, such as customer screening, decision-making processes, and records retention practices (Figure 1). Certification would likely thus selectively utilize specific components of a test suite in the testing process, tailored to the specific mandate of a given certification authority. A test suite, however, should strive to be comprehensive in order to uncover differences in screening system performance at the margins in complex or poorly annotated cases, as well as to help better define what constitutes a sequence of concern. This distinction lays the groundwork for a comprehensive exploration of the challenges and opportunities in advancing screening methodologies.

In the remainder of this article, we discuss (1) the specific methods by which the authors developed a prototype test suite in the form of a test dataset for selected controlled taxa and in methods for comparing qualitatively different screening tools; (2) the results produced by this comparison, highlighting both the level of consensus that was found among tools and the challenges remaining; and (3) conclusions that may be drawn from these results and their implications for the development of test suites, tools, standards, and policies.

## Methodology

The initiative to develop a standardized DNA screening test suite commenced in the summer of 2022, with a project scope workshop held in November 2022 to define the objectives and parameters of the undertaking.^11^ Subsequent to constructive feedback from workshop participants, a dedicated International Gene Synthesis Consortium (IGSC) working group was established and led to the formulation of a comprehensive proposal in January 2023 for a pilot project to develop a prototype test set.

**Figure 2.**
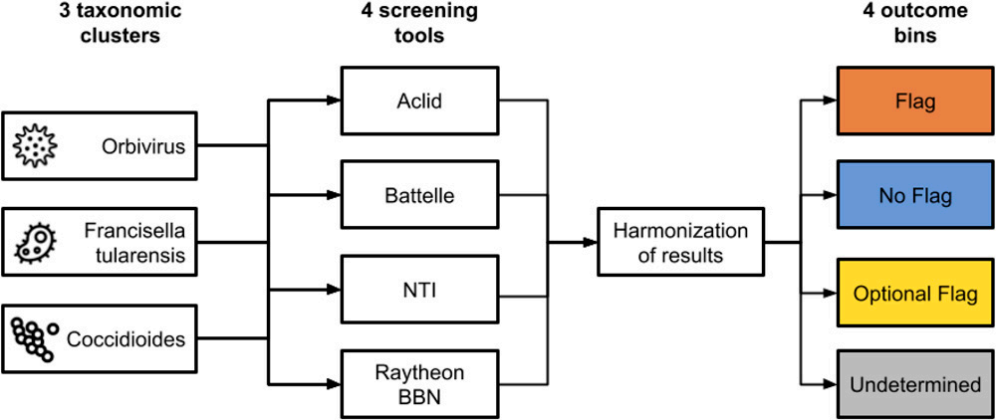
Prototype test set was developed by evaluating nucleic acid sequences from three taxonomic clusters with four screening tools and then harmonizing results to categorize them into four categories of screening outcome.

The development of the prototype test, executed between March and September 2023, involved the participation of six organizations, encompassing both tool providers (Aclid, Battelle, NTI, and Raytheon BBN) and synthesis companies (Twist and IDT) (Figure 2). Aclid’s platform brings customer screening and alignment-based sequence screening into a single platform along with automated and artificial intelligence (AI)-driven data curation. Battelle’s UltraSEQ^8^ uses an information-based alignment approach coupled with machine learning to identify taxonomic best matches and sequences of concern. The Common Mechanism uses alignment-based methods to identify taxonomic best matches and matches to benign synthetic biology parts and profile-based methods to identify toxins, virulence factors, and proteins with benign housekeeping functions (https://doi.org/10.1089/apb.2023.0034). FAST-NA Scanner uses Bloom filters to identify nucleic acid and amino acid k-mer “signatures” that are unique to specific types of pathogen or toxins, and then scans input sequences for these signatures.^6^ All of these tools evaluate sequences in all possible coding frames.

The prototype test set comprises sequences for three taxonomic clusters of organisms: *Francisella* (bacteria), *Orbiviruses* (virus), and *Coccidioides* (fungus) (Figure 2). The IGSC Regulated Pathogen Database currently identifies 39 taxonomic clusters covering the organisms listed by the Australia Group (AG) control lists, EU list of dual-use items, the United States Federal Select Agent Program Select Agent and Toxins List, and the United States Export Administration Regulation Commerce Control List. These three clusters were selected from the IGSC list of clusters on the basis that (1) they represent different kingdoms and (2) each cluster contains a small number of regulated organisms with minimal taxonomic ambiguity (unlike, e.g., the taxonomic ambiguities in the *Xanthomonadaceae* cluster or the large number of species in the *Mononegavirales* cluster). Sequence data were sourced from the National Center for Biotechnological Information nucleotide database, filtered based on belonging to the specified taxonomic clusters and falling within a sequence length range of 200–10,000 bases. In the case of *Coccidioides*, the number of sequences available was large and, thus, was capped at 10,000, randomly selected from all sequences matching the criteria. Each tool provider independently screened the sequences utilizing their default methodologies, and the outcomes were systematically collated.

The next step in test set development is to map the output of the tools onto a common framework for comparison, which is done by abstracting the outputs based on a workflow decision shared by all screening tools: “clearing” versus “flagging” sequences. For any sequence query, a screening tool must determine whether that sequence can be confidently “cleared”—that is, asserted to have no role in pathogenesis and not to be subject to control under any regulatory framework. If a sequence cannot be cleared, a screening tool can either declare that sequence as subject to specific regulatory control (“controlled”) or “flag” that sequence for further human expert review. Given the complexity of current regulatory frameworks, it is often very difficult for screening systems to confidently identify controlled sequences. Instead, systems generally clear or flag each sequence. Follow-up human review of flagged sequences then determines whether a sequence is ultimately controlled under a regulatory framework or not.

This cleared versus flagged dichotomy also introduces the first of many decision points faced by sequence screening implementations: Is a sequence guilty (“flagged”) until proven innocent? Or is that sequence innocent (“cleared”) until proven guilty? Which of these two worldviews the tools take can have a significant impact on the kinds of evidence and analysis required for triggering human review? Each of the four tools in this study had a different system for flagging sequences for human review, underpinned by different computational strategies for identifying sequences with potential for misuse, and different decision boundaries for flagging a sequence as potentially concerning.

To harmonize the diverse tool results, a mapping process was implemented, categorizing sequences into “Flag” (“guilty”), “No Flag” (“innocent”), or “Optional Flag” (“not guilty” or “not innocent”) (Figure 2). Subsequently, a comparative analysis was undertaken to identify consensus and conflicts between tool determinations. Sequences were designated to the final “Flag” category if flagged by at least one tool and not cleared by any tool. Conversely, sequences were allocated to the “No Flag” category if cleared by at least two tools and not flagged by any tool. “Optional Flag” sequences were not flagged or cleared by any tool but were found to be unique to a regulated pathogen. These sequences generally had unclear links to pathogenicity, either because of the indirect nature of evidence for potential contribution to pathogenicity or because of a lack of information about biological function. More conservative tools thus tended to classify these sequences as “not innocent” while more aggressively clearing tools tended to classify them as “not guilty.”

Conflicts, denoting instances in which at least one tool recommended “Flag” status while at least another recommended “No Flag,” were systematically identified and grouped based on gene annotation. The resolution of conflicts was achieved through discussion within the working group guided by, where available, annotated protein family relationships, experimental evidence, and published literature. In cases where either regulatory or biological uncertainty persisted after discussion, sequences were classified as “Undetermined” (Figure 2), signifying the need for further investigation or clarification. This methodological framework ensured a systematic and collaborative approach to categorizing sequences within the prototype test set, laying the groundwork for subsequent refinement and broader applications in the development of a DNA screening test suite that comprehensively spans the set of organisms and molecules controlled under existing regulatory frameworks.

## Results

### Classification of Test Sequences

**Figure 3.**
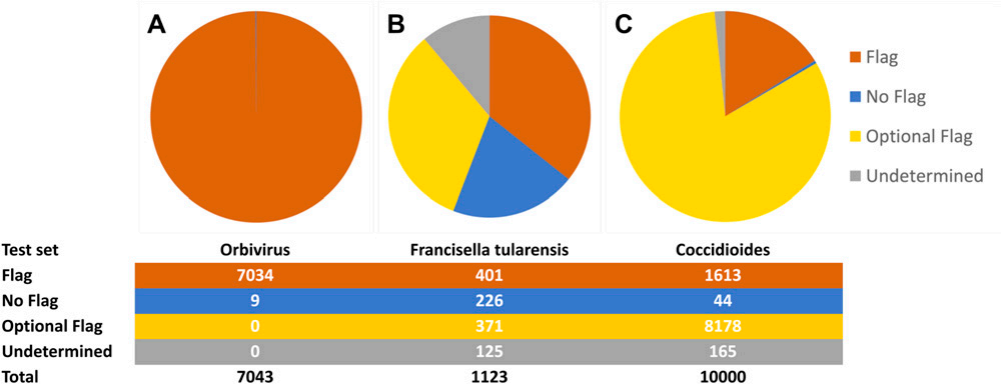
DNA screening classification distribution for the prototype test set across representative taxonomic clusters from the viral, bacterial, and fungal kingdoms.

The outcomes of the prototype test set exposed notable divergence in screening results across the three clusters under scrutiny. For the *Orbivirus* viral pathogen cluster, comprising African horse sickness virus and Bluetongue virus, agreement was universal, and nearly all sequences were flagged: 99.87% of *Orbivirus* test sequences were assigned a “Flag” status, barring a few exceptions (0.13%) that were cleared because of the sequence being mislabeled (e.g., AY397620.1) or notional (e.g., 1H1K_M) (Figure 3A).

In contrast, determinations for the bacterial pathogen *Francisella tularensis* exhibited a more even distribution across the four defined categories (Figure 3B). The four tools made consistent judgments for 88.9% of the test sequences. However, more than a third of the consistent judgments were “Optional Flag,” meaning that although the judgments were consistent (“not innocent” and “not guilty”), the actual regulatory status of the sequence is unclear. Conflicts between tools placed the remaining 125 sequences (11.1%) into the “Undetermined” category. Further analysis grouped these sequences into 18 clusters: 2 pathogenicity-linked genes with unsettled scientific questions regarding their significance, 13 conserved genes judged to have minimal risk but uncertain regulatory status (e.g., recombinase A, riboflavin biosynthesis protein), and 3 collections of heterogeneous, poorly annotated sequences (e.g., “regions of difference” and “microsatellites”).

For the *Coccidioides* fungal pathogen cluster, comprising *Coccidioides immitis* and *C. posadasii*, judgments were also highly consistent, with the tools producing consistent judgments for 98.4% of the test sequences (Figure 3C). In contrast to *Orbivirus* and *F. tularensis*, however, there were a much higher proportion of “Optional Flag” sequences: nearly 5/6 of all consistent judgments were in this category, underscoring the existing gaps in our understanding of the functional roles of eukaryotic genes, especially with regard to specific mechanisms of fungal pathogenicity. This observation reflects the inherent complexity associated with regulating and screening eukaryotic organisms, shedding light on the nuanced challenges that necessitate further exploration and refinement in the evolving landscape of DNA synthesis screening. Only 165 sequences had conflicts resulting in them being categorized as undetermined (1.7%). Most of these were poorly annotated heterogeneous sequences from cDNA libraries, with the remainder being either hypothetical proteins or conserved genes judged to have minimal risk but uncertain regulatory status.

## Discussion

In the process of developing the prototype test set, the working group identified a number of challenges that reflect the challenges posed by state-of-the-art DNA screening more broadly.

### Divergent Outputs in Screening Tools: Navigating Categorization Challenges

The outputs of each screening tool exhibited qualitative distinctions, introducing variability in the categorization of sequences. Some tools took a conservative approach (“guilty until proven innocent”), flagging orders containing sequences with potentially meaningful similarity to known regulated sequences. Conversely, other tools took a strict approach (“innocent until proven guilty”), flagging sequences for human attention if a sequence was most similar to a regulated pathogen sequence and could not be confidently cleared as exempt from regulatory controls. In addition, one tool used a tiered system with multiple classes corresponding to the “Flag,” “Optional Flag,” and “No Flag” designations used by other tools not incorporated in the alpha test.

These categorization differences reflect the diverse computational approaches used by various screening systems, as well as differences in the use cases that they are designed to support and differences in how conservatively their designers approach certain regulatory ambiguities. For example, users of screening software may have differing preferences, with some seeking minimal flags to reduce the cost associated with follow-up on flagged sequences. Conversely, other DNA providers prioritize minimizing the risk of providing potentially misusable DNA to customers and allocate additional resources to investigate orders where there is greater uncertainty regarding the regulatory status of the sequence. These decisions (balancing cost with risk) stem from how one interprets the screening guidance and regulatory documents, which leave several technical areas unclear (e.g., the 2023 U.S. Department of Human and Health Services [HHS] guideline’s definitions of Sequences of Concern). Differences in organization scale and business model are significant as well: for example, the needs of a large DNA provider making vast numbers of short constructs at low margin are quite different than those of a small DNA provider making small numbers of high-value constructs for a small number of customers.

Complicating matters, a lack of clarity from regulators on how to treat “Optional Flag” and “Undetermined” sequences introduces ambiguity in the practical implementation of screening guidance. Addressing these categorization challenges requires careful consideration of user preferences, potential misuse risks, and regulatory guidance to ensure effective and transparent implementation of DNA synthesis screening protocols.

### Navigating Scientific Uncertainties in Pathogen Genes

Considerable scientific uncertainty surrounds the role of many genes within pathogen genomes concerning the process of causing disease. Complicating matters further, there is significant overlap between genes required for causing disease in pathogens and those necessary for close association with a host in nonpathogenic microorganisms. Examples include genes involved in adhering to host cells or suppressing host immune response.

This inherent uncertainty is reflected in the test set classification of sequences as “Undetermined.” Some tools identify these sequences as shared by a broad range of nonpathogenic organisms, whereas others implicate them in the process of causing disease in pathogens based on experimental survey results. This uncertainty is poised to escalate significantly with the new guidance from the HHS,^4^ which recommends developing best practices to expand the definition of sequences of concern to include any genes involved in the pathogenesis of serious disease or toxicity, even when they are from unregulated agents. Addressing this uncertainty is critical, especially in the context of evolving guidelines, and will require careful consideration of the potential implications of expanding the scope of sequences of concern.

### Navigating Uncertainties in Sequence Categorization

As part of the development of the prototype test set, a considerable fraction of bacterial and fungal sequences were categorized as “Optional Flag,” comprising sequences unique to a regulated pathogen but without either a clear link to pathogenicity or a known function deemed sufficiently distant from pathogenesis to allow them to be cleared. This finding indicates that none of the screening tools used in testing identified a connection between these sequences with disease or toxicity (“guilty”) nor an essential benign housekeeping function (“innocent”). However, this classification does not eliminate the possibility that a thorough investigation into the genes encoded by the sequence, and their documentation in scientific literature, could eventually reveal a known role in pathogenicity. The challenge lies in the inefficient translation of scientific discoveries about pathogenicity genes to curated databases and the requirement for synthetic DNA manufacturers to make a decision at the time of order using the best available information.

Conducting a manual investigation of each sequence can take several hours per sequence and requires knowledge of microbiology, biochemistry, and toxicology. Such investigation is impractical for the number of results in the prototype test set. Scaling this process to a more extensive dataset would pose an even greater challenge. The submission of these uncertain cases to regulatory authorities for export control classification introduces additional delays and, given the sheer number of “Optional Flag” sequences even in this prototype test set, risks overwhelming government classification authorities. Addressing this challenge in the context of universalizing DNA synthesis screening requires innovative approaches to evaluating orders and to regulating the consequences of those evaluations.

Engagement with country-specific export control authorities can take the form of individual license applications for approval to export a sequence or broader advisory opinions on interpretation of regulatory language that can be shared. Notably, today there is no mechanism for sharing the outcomes of individual export control license requests within the DNA screening community. An effective solution to these challenges involves incorporating past and future decisions as additional inputs to help decide how to categorize sequences that are currently in the “optional flag” or “undetermined” categories. This approach holds promise for resolving uncertainties and minimizing duplication of efforts in the review of classification requests, streamlining the universal adoption of DNA synthesis screening.

### Source Data Quality Challenges in DNA Sequence Screening

Another key methodological challenge that makes it difficult to establish a single, reliable test set for nucleic acid screening is quality control issues in publicly available databases, from which test data are drawn or to which test data are compared. These databases were not originally designed with regulatory controls in mind. Submitted sequences can be inaccurately labeled with the incorrect source organism. This can happen because of human error during submission; contamination during the sample collection, preparation, or sequencing process; imperfections in the automation of sequence submission; or even deliberate misbehavior such as plagiarism, scientific fraud, or IP obfuscation. Many sequences also include chimeric material from biotechnological tools such as purification tags, reporters, and delivery vectors, which can also result in incorrect classifications of sequences.^12^ Correcting or mitigating the impact of these issues in public annotation sources poses a significant challenge in enhancing the precision of DNA screening processes.

## Conclusions and Future Directions

The prototype phase of our study demonstrated a high degree of consistency in the classification decisions made by current DNA synthesis screening tools. At the same time, it has also shed light on additional challenges to be overcome in developing a comprehensive DNA sequence screening test set, reflecting broader issues in the domain of regulatory control of DNA sequences and risk estimation of stand-alone biological components. Our comparison of four existing screening tools has highlighted both the current efficacy of those tools and uncertainties and technical complexities in confidently assessing regulatory restrictions and the potential for misuse associated with DNA sequences.

An enduring challenge faced by DNA synthesis screening systems is how to make high-stakes decisions with low information. Clearing a nucleic acid sequence for synthesis and shipment that requires an export license can violate both export control laws and regulations on domestic possession, thereby resulting in severe fines and other legal penalties, along with reputational damage.^13^ However, if the false-positive rate of a screening system is too high, users of the software will face a significant and ongoing cost because of the time required to investigate and clear unnecessary flags. High false positives also increase the risk of decision fatigue that causes genuinely dangerous sequences to be accidentally cleared.

To facilitate consistent, low-cost, global-scale implementation of DNA screening, there is a pressing need to scale up this test set creation process to cover all organisms included in the IGSC Regulated Pathogen Database. Governments should also explore more efficient approaches to scaling DNA-based commodity classification requests. Addressing the challenges identified during the construction of the prototype test set, particular attention must be given to achieving clarity in flagging sequences otherwise categorized as “Undetermined” or “Optional,” recognizing the unique challenges posed by these categories for less-well-annotated bacteria and eukaryotes, respectively.

Achieving international harmonization, especially within multilateral arrangements such as the AG, is paramount to ensure a uniform global capability to detect sequences with potential for misuse in synthetic nucleic acid order streams. In the next steps of our initiative, we plan to request that the U.S. Commerce Department Bureau of Industry and Security (BIS) carry out commodity classifications for “Undetermined” genes identified to date and conduct a comprehensive tool comparison for all taxonomic groups covered by international control lists. Coordination will also be needed with NIST pursuant to their charge under the Executive Order on AI and with other relevant national and international organizations. Furthermore, expanding the involvement of additional tools and nongovernmental participants in the working group will contribute to a more robust and diverse perspective.

Looking ahead, the curation of a full-scale test set is recommended. This set would include all 39 threat clusters in the IGSC Regulated Pathogen Database. The Regulated Pathogen Database is based on the organisms marked as controlled under the AG multilateral regime. For each cluster, the test set would incorporate both DNA and protein sequences and consider both controlled species and noncontrolled “cousin” species, resulting in a total of 156 test sets. For clusters with many sequences available, there are also likely better approaches to selecting a subset than the random sampling used in this test for *Coccidioides*. This work will require collaborative efforts with BIS and other AG member governments to address “bulk” classification challenges as well as to determine how governments can address requests to classify sequences for which little to no public annotation exists. Future work will also explore the robustness of screening systems to challenging cases, such as obfuscated, fragmented, or divergent sequences.

The revised HHS Screening Framework Guidance for Providers and Users of Synthetic Nucleic Acids has also recommended substantially expanding the scope of screening to include “sequences known to contribute to pathogenicity or toxicity, even when not derived from or encoding regulated biological agents” as soon as it is practical to implement this.^4^ Determining which sequences meet this expanded inclusion criteria poses a major challenge to the development of both the screening tools and the methods to test them. Expanding our test dataset to include such sequences will be an important future challenge that will benefit from coordination and shared effort between screening providers. Ultimately, the prediction of function from sequence alone using technologies such as large language models and other forms of neural networks may be necessary to create a comprehensive screening methodology that is less reliant on human interpretation.^15–17^ By addressing these critical aspects, we aim to refine and advance our screening processes, ensuring a more robust and effective approach to DNA synthesis screening on a global scale.
